# Clodronate, an inhibitor of the vesicular nucleotide transporter, ameliorates steatohepatitis and acute liver injury

**DOI:** 10.1038/s41598-021-83144-w

**Published:** 2021-03-04

**Authors:** Nao Hasuzawa, Keita Tatsushima, Lixiang Wang, Masaharu Kabashima, Rie Tokubuchi, Ayako Nagayama, Kenji Ashida, Yoshihiro Ogawa, Yoshinori Moriyama, Masatoshi Nomura

**Affiliations:** 1 Division of Endocrinology and Metabolism, Department of Internal Medicine, Kurume University School of Medicine, 67 Asahi-machi, Kurume, 830-0011 Japan; 2 Department of Medicine and Bioregulatory Science, Graduate School of Medical Sciences, Kyushu University, Fukuoka, 812-8582 Japan; 3 Department of Psychosomatic Medicine, Graduate School of Medical Sciences, Kyushu University, Fukuoka, 812-8582 Japan; 4 Department of Chemistry, Kurume University School of Medicine, Kurume, 830-0011 Japan

**Keywords:** Drug discovery, Endocrinology, Gastroenterology, Molecular medicine

## Abstract

The vesicular nucleotide transporter (VNUT) is responsible for the vesicular storage and release of ATP from various ATP-secreting cells, and it plays an essential role in purinergic signaling. Although extracellular ATP and its degradation products are known to mediate various inflammatory responses via purinoceptors, whether vesicular ATP release affects steatohepatitis and acute liver injury is far less understood. In the present study, we investigated the effects of clodronate, a potent and selective VNUT inhibitor, on acute and chronic liver inflammation in mice. In a model of methionine/choline-deficient diet-induced non-alcoholic steatohepatitis (NASH), the administration of clodronate reduced hepatic inflammation, fibrosis, and triglyceride accumulation. Clodronate also protected mice against high-fat/high-cholesterol diet-induced steatohepatitis. Moreover, prophylactic administration of clodronate prevented d-galactosamine and lipopolysaccharide-induced acute liver injury by reducing inflammatory cytokines and hepatocellular apoptosis. In vitro, clodronate inhibited glucose-induced vesicular ATP release mediated by VNUT and reduced the intracellular level and secretion of triglycerides in isolated hepatocytes. These results suggest that VNUT-dependent vesicular ATP release plays a crucial role in the recruitment of immune cells, cytokine production, and the aggravation of steatosis in the liver. Pharmacological inhibition of VNUT may provide therapeutic benefits in liver inflammatory disorders, including NASH and acute toxin-induced injury.

## Introduction

Non-alcoholic fatty liver disease (NAFLD) is highly prevalent in all regions of the world and a growing public health problem^1^. NAFLD represents a spectrum of liver disease ranging from simple steatosis to pathologically more severe forms, such as non-alcoholic steatohepatitis (NASH) and cirrhosis. NAFLD is slowly progressive, but in certain cases, it progresses rapidly. It is reported that the progression of each fibrosis stage takes 14.3 years in patients with NAFLD and 7.1 years in those with NASH^2^. Liver fibrosis is considered the most important predictor of mortality in NAFLD, as the risks of liver-related and all-cause mortality increase exponentially with each fibrosis stage^3^. Thus, therapeutic approaches for the prevention of disease progression are urgently needed. However, limited treatment options are currently available for NAFLD and its pathogenesis has not yet been fully elucidated.

NASH is defined as the presence of steatosis and inflammation, and the latter is the main independent risk factor for fibrosis progression^4^. Among the endogenous triggers of liver inflammation, extracellular ATP and its degradation products, such as ADP and adenosine, have been demonstrated to be important danger signals that mediate a wide spectrum of pathological processes in the liver^5^. Liver cells express various purinergic receptor subtypes^6^. For instance, purinergic receptor P2RX7, a key player in inflammation, was shown in rodents to be involved in alcohol- or diet-induced steatohepatitis, as well as acetaminophen hepatotoxicity^7–9^. Recently, it was reported that purinergic receptor P2RX7 is expressed by infiltrating monocytes and resident Kupffer cells in the livers of NASH-affected individuals^10^. Extracellular nucleotides are released from liver cells through three pathways: simple leakage via cellular breakage, permeation through ATP-permeable channels in the plasma membrane such as connexin hemichannels and pannexin channels, and exocytosis (vesicular ATP release)^6^. A recent study by Vinken and colleagues showed that connexin hemichannels are involved in ATP release from hepatocytes, and the inhibition of these hemichannels alleviates choline-deficient, high-fat diet-induced NASH in mice^11^. They further demonstrated that the genetic depletion of pannexin 1 protected mice from acetaminophen-induced acute liver failure and diet-induced NASH^12^. These studies suggest that not only purinergic signal receptors but also nucleotide release pathways are deeply involved in the progression of acute and chronic liver diseases.

Vesicular nucleotide transporter (VNUT) is responsible for the vesicular storage and release of ATP and plays an essential role in purinergic signal transmission^13,14^. In *Vnut* knockout (*Vnut*^*−/−*^) mice, ATP-secreting cells such as neurons, epithelial cells, and immune cells lack the capacity to release vesicular ATP. Furthermore, *VNUT*^*−*/*−*^ mice exhibit attenuated pain perception, reduced inflammation, and increased insulin sensitivity^15,16^. We previously found that clodronate, a first-generation bisphosphonate, is a specific inhibitor of VNUT, preventing vesicular ATP release and resulting in the blockade of purinergic chemical transmission in vivo^14,16–19^. Remarkably, the mode of action of clodronate is totally different from that of clodronate encapsulated in liposomes used for macrophage depletion. As revealed by in vitro assays, clodronate itself directly, selectively, and strongly inhibits VNUT with a half-maximal inhibitory concentration (IC_50_) value of 15.6 nM^16,19^.

Recently, we found that hepatocytes also express VNUT and secrete ATP through a VNUT-mediated mechanism upon glucose stimulation, and this ATP secretion does not occur in the hepatocytes of *Vnut*^*−/−*^ mice^20^. Furthermore, *Vnut*^*−/−*^ mice are protected from diet-induced steatohepatitis and fibrosis, which suggests that VNUT is involved in pathological conditions of the liver. These findings led us to explore whether clodronate could be used to treat acute and chronic liver diseases. In the present study, therefore, we examined the effect of clodronate administration on diet-induced NASH in mice. We also investigated whether clodronate is effective for d-galactosamine (GalN)- and lipopolysaccharide (LPS)-induced acute liver injury.

## Results

### Pharmacologic inhibition of vesicular ATP release ameliorates MCD diet-induced steatohepatitis

**Figure 1:**
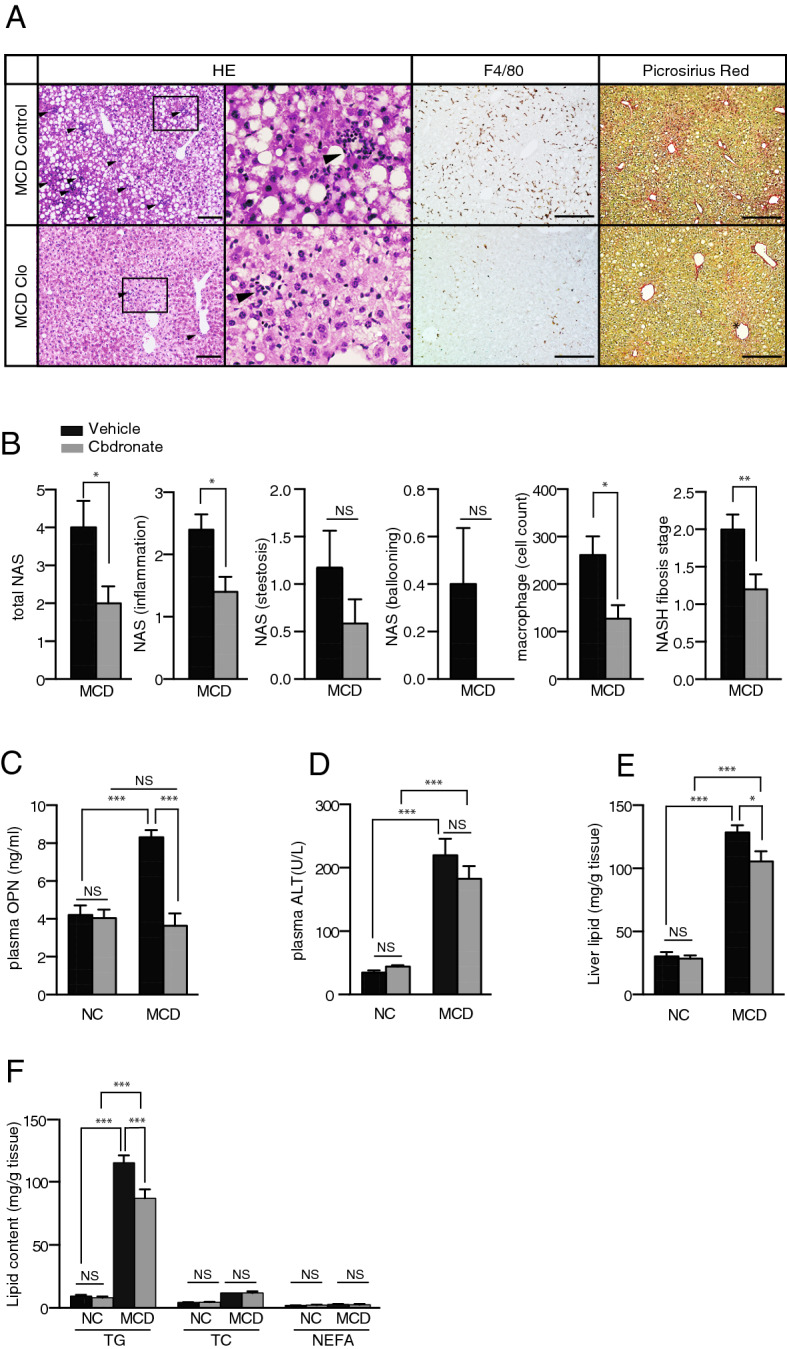
Pharmacologic inhibition of ATP signaling by clodronate protects against methionine- and choline-deficient diet-induced steatohepatitis. Ten-week-old C57BL/6 male mice were fed with normal chow (NC) or a methionine- and choline-deficient (MCD) diet for 4 weeks. The vehicle or 20 mg/kg body weight clodronate (Clo) was administered via daily subcutaneous injection for 4 weeks (n = 5–10 MCD diet-fed, per treatment; n = 3–5 NC-fed, per treatment). (**A**) Hematoxylin and eosin (HE) staining (arrowheads indicate inflammatory foci), F4/80 immunostaining, and Picrosirius Red staining of paraffin-embedded liver tissue from MCD diet-fed mice treated with the vehicle or clodronate. Scale bars, 100 µm. (**B**) The total NAFLD activity score (NAS); the individual scores for lobular inflammation, steatosis, and ballooning; the numbers of macrophages based on a count of the F4/80-positive cells per high-power field; and the NASH fibrosis stage were quantified in the MCD-fed groups. (**C**) The plasma osteopontin level was quantified with an enzyme-linked immunosorbent assay. (**D**) The plasma ALT activity was quantified. (**E**) The total hepatic lipid content was determined using Folch’s method. (**F**) The hepatic levels of triglycerides (TG), total cholesterol (TC), and nonesterified fatty acids (NEFA) were assessed with enzyme assays. All data are presented as the mean ± S.E.M. *P < 0.05, **P < 0.01, ***P < 0.001. *NS* not significant, *ALT* alanine aminotransferase.

Purinergic signaling is involved in hepatic inflammation and fibrosis, two of the major pathological features of NASH^5^. To investigate the effect of vesicular ATP release on these pathologies, we used a mouse model of NASH induced by a methionine- and choline-deficient (MCD) diet. Ten-week-old C57BL/6 male mice were fed an MCD diet with daily subcutaneous injections of the vehicle or clodronate (20 mg/kg/day). MCD diet-induced body weight loss and organ-to-body weight ratios were not altered by clodronate treatment (see Supplementary Fig. S1 online). After 4 weeks, we observed that clodronate dramatically protected the mice against MCD diet-induced liver inflammation and fibrosis as indicated by their improved histology. As shown in Fig. 1A, hematoxylin–eosin (HE) staining demonstrated that intralobular inflammatory foci, which are characteristic of NASH, were frequently observed in the livers of the MCD group but not in the clodronate-treated livers. The NAFLD activity score, particularly the lobular inflammation score component, showed that clodronate ameliorated inflammation and liver damage (Fig. 1B). The numbers of F4/80-positive macrophages were also significantly reduced in this group (Fig. 1A,B). Further, Picrosirius Red staining revealed reduced fibrosis progression in the clodronate-treated group as evidenced by the NASH fibrosis staging (Fig. 1A,B). Consistent with this, the MCD diet induced increases in plasma osteopontin, a pro-inflammatory cytokine promoting liver fibrosis, and this effect was completely prevented by clodronate treatment (Fig. 1C). Plasma ALT levels were similar between the vehicle- and clodronate-treated groups, both for the normal diet-fed and MCD diet-fed groups (Fig. 1D). Interestingly, treatment with clodronate ameliorated not only inflammation and fibrosis, but also hepatic steatosis (Fig. 1E). Lipid analysis revealed that the liver triglyceride contents were significantly reduced in the clodronate-treated group (Fig. 1F).

**Figure 2:**
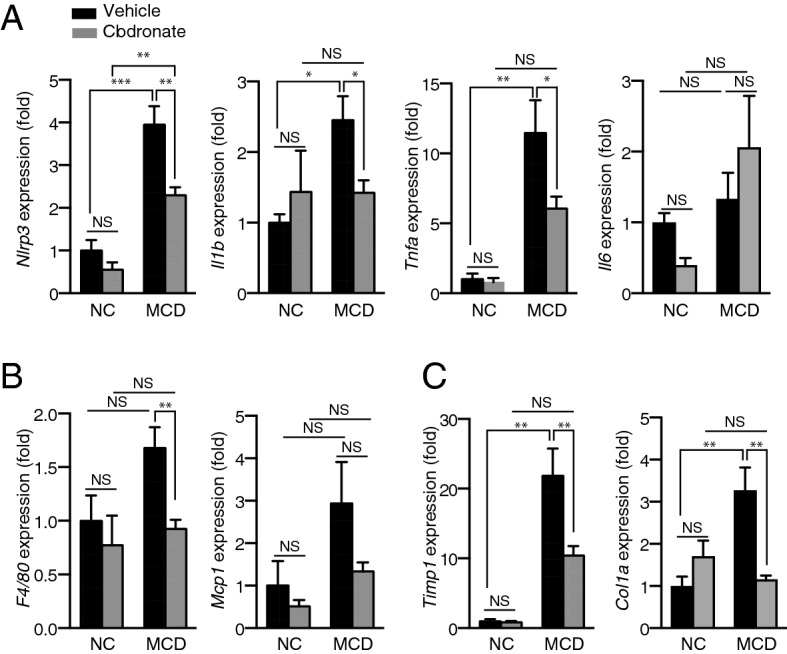
Clodronate suppresses inflammatory gene expression in the livers of mice fed the methionine- and choline-deficient diet. The livers of mice treated as described in Fig. 1 were analyzed by qRT-PCR [n = 10, methionine- and choline-deficient (MCD) diet-fed, per treatment; n = 3–5, normal chow (NC)-fed, per treatment]. (**A**) Relative mRNA levels of the inflammatory genes *Nlrp3*, *Il1β*, *Tnfa*, and *Il6*. (**B**) Relative mRNA levels of the macrophage marker *F4/80* and chemokine *Mcp1*. (**C**) Relative mRNA levels of the fibrosis-related genes *Timp1* and *Col1a1*. The data are shown as the fold change relative to the vehicle-treated group fed with the NC diet. All data are shown as the mean ± S.E.M. *P < 0.05, **P < 0.01, ***P < 0.001. *NS* not significant.

The ameliorated steatohepatitis was accompanied by reduced inflammatory gene expression as analyzed by quantitative RT-PCR (qRT-PCR). Clodronate-treated mice demonstrated significant protection from the upregulation of *Nlrp3*, *Il1β*, and *Tnfα* (but not *Il6*) induced by the MCD diet (Fig. 2A). In concert with the reduced numbers of infiltrating macrophages, we observed decreased expression of *F4/80* mRNA and a trend toward the reduced expression of *Mcp1* (Fig. 2B) in MCD diet-fed mice treated with clodronate compared with the vehicle-treated control. This inhibition of cytokine gene expression was not accompanied by a reduction in the protein levels of Il1β, Tnfα, or Mcp1 in the liver or plasma (see Supplementary Fig. S1 online). This is consistent with a previous report stating that TNFα protein levels in the liver only increase up to 2 weeks after initiating the MCD diet, while *Tnfa* mRNA expression, as well as osteopontin protein levels, increase after 4 weeks of MCD diet treatment^21^. Hepatic mRNA levels of the fibrosis markers, *Timp1* and *Col1a1*, revealed the protective effect of clodronate against MCD diet-induced liver fibrosis (Fig. 2C). Taken together, these data suggest that vesicular ATP release promotes inflammation and fibrosis, as well as the development of steatosis in MCD diet-induced NASH.

### Oral administration of Clodronate prevents high-fat, high-cholesterol diet-induced steatohepatitis

**Figure 3:**
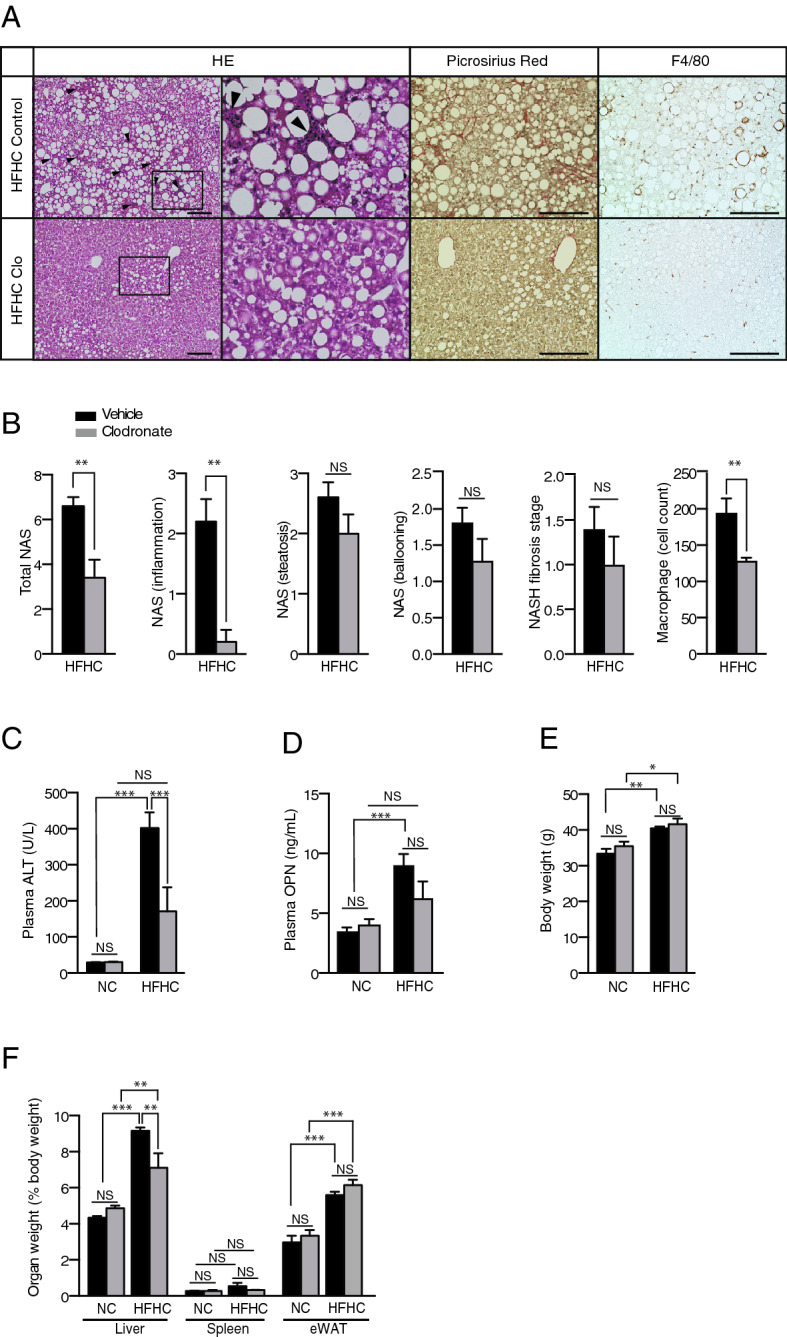
Oral administration of clodronate prevents high-fat, high-cholesterol diet-induced steatohepatitis. Ten-week-old C57BL/6 male mice were fed a normal chow (NC) diet or a high-fat, high-cholesterol (HFHC) diet for 24 weeks. The vehicle or clodronate was administered in the drinking water (30 mg/kg body weight/day) for 24 weeks, n = 5 per treatment group. (**A**) Hematoxylin and eosin (HE) staining (arrowheads indicate inflammatory foci), Picrosirius Red staining, and F4/80 immunostaining of paraffin-embedded mouse liver tissue. Scale bars, 100 µm. (**B**) The total NAFLD activity score (NAS); the individual scores for lobular inflammation, steatosis, and ballooning; the NASH fibrosis stage; and the number of macrophages based on a count of the F4/80-positive cells per high-power field were quantified for the HFHC diet-fed mice. (**C**) Plasma ALT activity was determined by enzyme assay. (**D**) Plasma osteopontin (OPN) levels were quantified with an enzyme-linked immunosorbent assay. (**E**) Bodyweight, and (**F**) organ weight expressed as a percentage of the body weight, were determined at the end of the study. All data are presented as the mean ± S.E.M. *P < 0.05, **P < 0.01, ***P < 0.001. *NS* not significant, *ALT* alanine aminotransferase, *eWAT* epididymal white adipose tissue.

The fact that clodronate ameliorated the accumulation of lipids, as well as inflammation and fibrosis, led us to evaluate the contribution of VNUT to lipid metabolism in a more relevant physiological context. To this aim, we used a mouse NASH model induced by a high-fat, high-cholesterol (HFHC) diet^22^. Mice were fed normal chow (NC) or the HFHC diet for 24 weeks with or without the administration of clodronate (30 mg/kg/day) in the drinking water. We observed that the long-term oral administration of clodronate significantly protected against HFHC diet-induced steatohepatitis. Clodronate provided a high level of protection from NASH as indicated by liver histology and quantified by the NAFLD activity score (Fig. 3A,B). There was also a trend toward the reduction of fibrosis as evaluated by Picrosirius Red staining and NASH fibrosis staging (Fig. 3A,B), as well as *Timp1* mRNA expression (see Supplementary Fig. S2 online), in the livers of clodronate-treated mice. F4/80 staining revealed a significant decrease in mean hepatic macrophage number in HFHC diet-fed mice treated with clodronate compared with the control group (Fig. 3A). Unlike with the MCD diet, long-term HFHC diet administration did not significantly increase the gene expression of *Nrlp3*, *Il1β*, or *Tnfα* (see Supplementary Fig. S2 online). The protein levels of IL1β, TNFα, and MCP1 in the liver and plasma were similar between the vehicle- and clodronate-treated mice (see Supplementary Fig. S2 online). Plasma ALT was significantly reduced (Fig. 3C) and plasma osteopontin showed a trend toward reduction in the clodronate-treated group compared with the HFHC diet-fed control (Fig. 3D). The HFHC diet resulted in similar levels of body weight gain in both groups (Fig. 3E). However, the relative liver weight was significantly lower in the clodronate-treated group, suggesting ameliorated steatosis (Fig. 3F).

**Figure 4:**
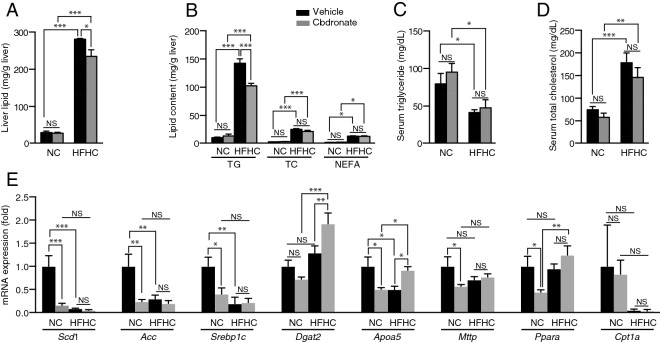
Clodronate suppresses triglyceride accumulation and de novo fatty acid synthesis-related gene expression in the liver. The lipid metabolism of mice treated as described in Fig. 3 was investigated (n = 5 per treatment group). (**A**) The total hepatic lipid content was determined using Folch’s method. (**B**) The hepatic levels of triglycerides (TG), total cholesterol (TC), and nonesterified fatty acids (NEFA) were assessed with enzymatic assays. (**C**) The serum triglyceride and (**D**) TC levels were quantified using biochemical assays. (**E**) The mRNA levels of genes associated with lipid metabolism in the livers of normal chow (NC) diet-fed mice and high-fat, high-cholesterol (HFHC) diet-fed mice were determined by quantitative real-time PCR. Gene expression is shown as the fold change relative to the vehicle-treated group fed the NC diet. All data are shown as the mean ± SEM. *P < 0.05, **P < 0.01, ***P < 0.001. *NS* not significant.

Liver lipid analysis revealed that treatment with clodronate attenuated the HFHC diet-induced accumulation of lipids (Fig. 4A), among which the triglyceride content was significantly reduced (Fig. 4B). The HFHC diet significantly decreased the serum triglyceride level and increased the total serum cholesterol compared with the NC diet (Fig. 4C,D), as reported previously^22^, and these changes were not affected by clodronate treatment. To investigate the underlying mechanism involved, lipid metabolism-related gene expression was analyzed. In the livers of NC diet-fed mice, the oral administration of clodronate significantly suppressed the expression of de novo fatty acid (FA) synthesis-related genes including *Scd1*, *Acc*, and *Srebp1c* (Fig. 4E). The expression levels of *Mttp*, a gene involved in very low density lipoprotein (VLDL) production and secretion, as well as *Apoa5*, which encodes a protein that facilitates the catabolism of triglyceride-rich lipoproteins in the plasma and the accumulation of intracellular triglycerides in the liver^23,24^, were also significantly reduced by clodronate treatment in NC-fed mice. *Pparα*, which encodes a transcriptional activator of genes involved in β-oxidation, was also significantly reduced by clodronate treatment in NC diet-fed mice, although this was not accompanied by changes in *Cpt1a*, which encodes an enzyme catalyzing the import of fatty acid into the mitochondria. The HFHC diet strongly suppressed the FA synthesis-related genes *Scd1*, *Acc*, and *Srebp1c* and this was not affected by clodronate treatment. Conversely, the gene expression of *Dgat2*, a triglyceride synthesis-related gene, and that of *Apoa5* was significantly increased by clodronate treatment compared with the vehicle-treated control HFHC diet-fed mice. *Ppara,* but not *Cpt1a*, was significantly upregulated by HFHC feeding in the clodronate-treated group but not in the vehicle-treated group, which might in part have contributed toward the attenuation of steatosis by activating β-oxidation.

### Clodronate protects against d-galactosamine/lipopolysaccharide-induced liver injury

**Figure 5:**
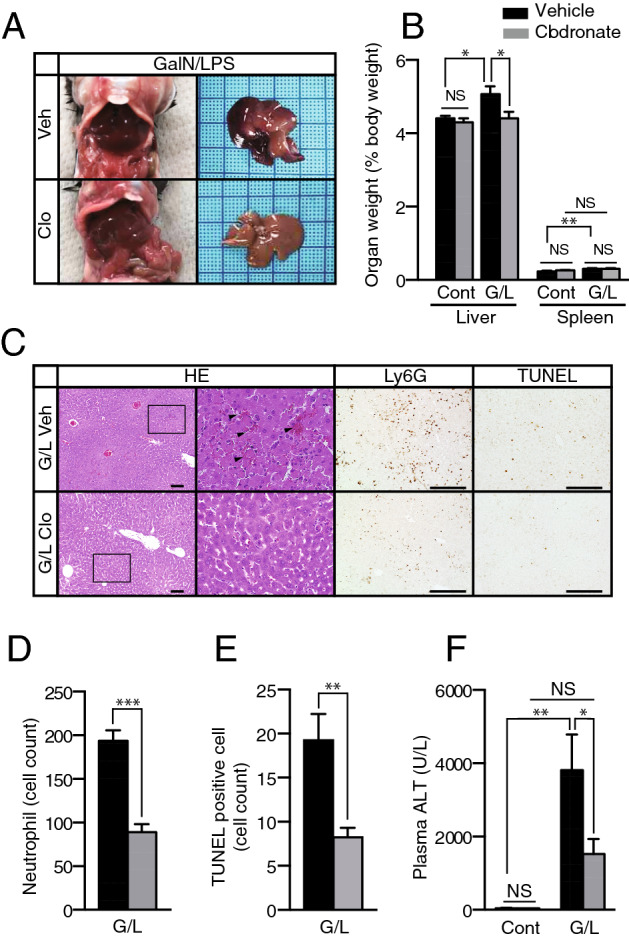
d-Galactosamine- and lipopolysaccharide-induced liver injury was prevented by pre-treatment with clodronate. Liver injury was induced in 10-week-old C57BL/6 male mice by intraperitoneal injections of 700 mg/kg of d-galactosamine (GalN) and 100 µg/kg of lipopolysaccharide (LPS). Mice were pretreated with the saline vehicle (Veh) or 50 mg/kg of clodronate (Clo) 1 h before GalN/LPS or control (saline) administration (n = 6 for the GalN/LPS administration group, per treatment; n = 3 for the control group, per treatment). (**A**) Representative liver morphology of mice, pre-treated with vehicle or clodronate, 6 h after GalN/LPS administration. (**B**) Organ weight expressed as a percentage of the bodyweight for each treatment group. (**C**) Hematoxylin and eosin (HE) staining (arrowheads indicate parenchymal hemorrhage), Ly6G immunostaining for neutrophils, and TUNEL staining for apoptotic cells. Scale bars, 100 µm. (**D**, **E**) Numbers of Ly6G-positive cells (**D**), and TUNEL-positive cells (**E**), per high-power field. (**F**) Plasma ALT levels were also evaluated using an enzyme assay. All data are shown as the mean ± S.E.M. *P < 0.05, **P < 0.01, ***P < 0.001. *Clo* clodronate, *Veh* saline vehicle, *NS* not significant, *ALT* alanine aminotransferase, *Cont* control (saline), *G/L* d-galactosamine and lipopolysaccharide treatment.

Clodronate is reported to have anti-inflammatory effects on immune cells such as macrophages and neutrophils through the inhibition of VNUT-dependent vesicular ATP release^16,25^. To test whether clodronate could exert beneficial effects on acute toxic liver injury, 10-week-old C57BL/6 male mice were subjected to intraperitoneal injections of d-galactosamine (GalN) and lipopolysaccharide (LPS). Mice were pretreated with the saline vehicle or 50 mg/kg of clodronate 1 h before GalN/LPS administration. At 6 h after GalN/LPS injection, the livers showed signs of congestion and significant increases in weight, while these changes were apparently prevented by pre-treatment with clodronate (Fig. 5A,B). Treatment with clodronate ameliorated GalN/LPS-induced histological changes in the liver based on HE staining, including the levels of parenchymal hemorrhage and inflammatory cell infiltration (Fig. 5C). Ly6G immunostaining and terminal deoxynucleotidyl transferase-mediated dUTP nick end labeling (TUNEL) staining confirmed reduced numbers of infiltrating neutrophils and apoptotic cells, respectively, in the livers of mice treated with clodronate (Fig. 5C–E). The attenuation of liver injury was further evidenced by the decreased serum levels of ALT in the clodronate-treated group (Fig. 5F).

**Figure 6:**
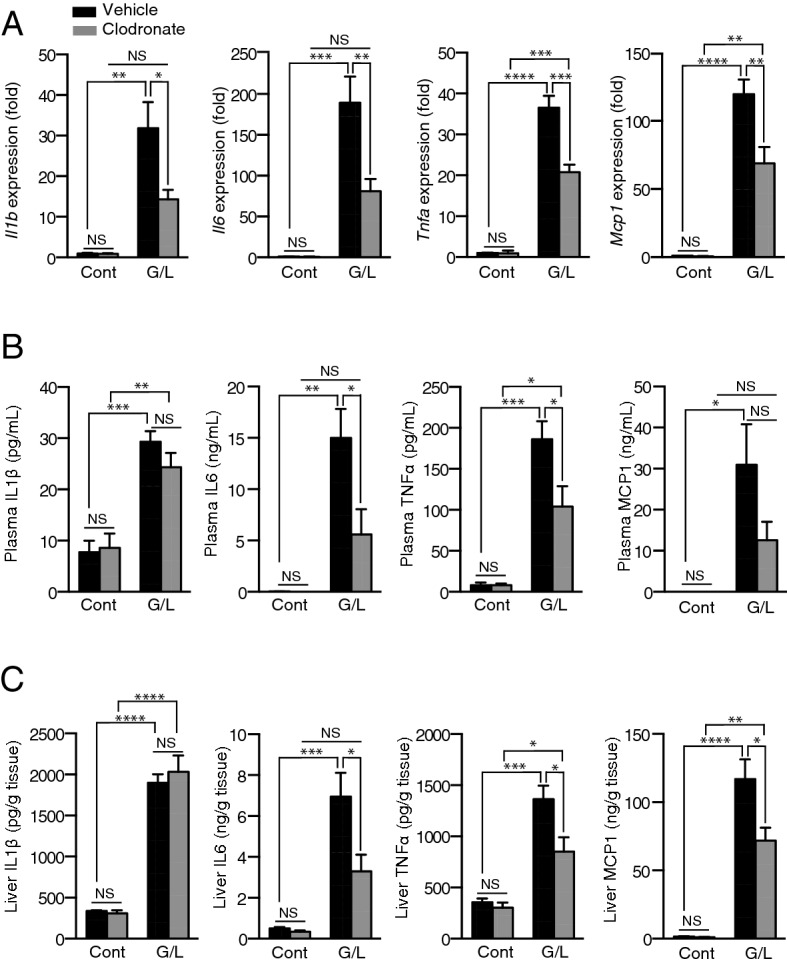
Clodronate suppresses inflammatory response in the plasma and liver of GalN/LPS-treated mice. The liver and plasma of mice treated as described in Fig. 6 were analyzed (n = 6 for the GalN/LPS administration group, per treatment; n = 3 for the control group, per treatment). (**A**) Hepatic mRNA levels of the inflammatory genes *Il1β*, *Il6*, *Tnfα*, and *Mcp1* were determined by qRT-PCR. (B, C) Plasma levels (**B**) and hepatic contents (**C**) of IL1β, IL6, TNFα, and MCP1 were determined using the BD Cytometric Bead Array. All data are shown as the mean ± S.E.M. *P < 0.05, **P < 0.01, ***P < 0.001. *NS* not significant, *Cont* control (saline), *G/L* d-galactosamine and lipopolysaccharide treatment.

Inflammatory gene expression in the liver was analyzed by qRT-PCR. At 6 h after GalN/LPS administration, the clodronate-treated mice exhibited approximately two-fold lower mRNA levels of *Il1β*, *Il6*, *Tnfα*, and *Mcp1* compared with the saline-treated mice (Fig. 6A). In concert with the mRNA findings, clodronate administration significantly reduced the protein levels of IL6 and TNFα in the plasma (Fig. 6B), and IL6, TNFα, and MCP1 in the liver (Fig. 6C), whereas clodronate treatment had no effect on IL1β. Extracellular ATP is known to induce inflammasome activation via P2X7 receptors and, thereby, IL-1β maturation and secretion^26^. However, our results suggested that in this model, clodronate affected inflammasome-independent pathways of inflammation.

### Effect of clodronate on inflammatory gene expression, ATP secretion, and triglyceride metabolism in cultured mouse primary hepatocytes

**Figure 7:**
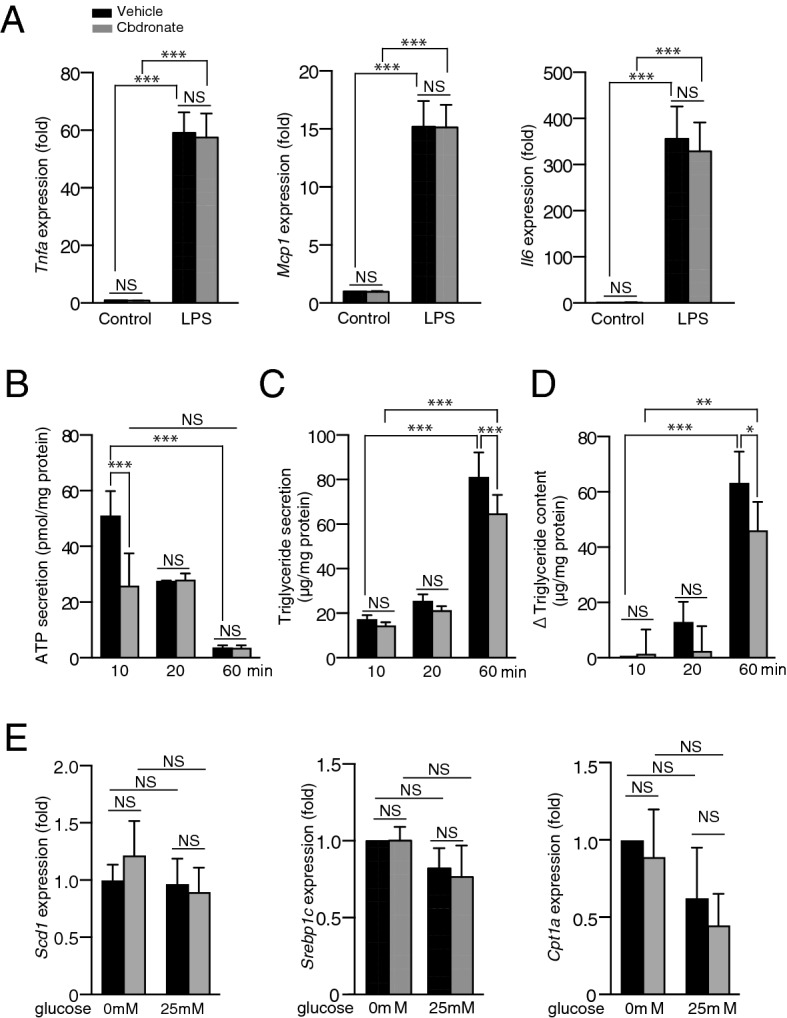
Clodronate inhibits ATP release and triglyceride synthesis but not inflammatory gene expression in mouse primary hepatocytes. Primary hepatocytes were isolated from 10-week-old C57BL/6 male mice. (**A**) Primary mouse hepatocytes were pre-incubated in the presence or absence of 10 µM clodronate for 30 min, followed by stimulation with 1 µg/mL lipopolysaccharide (LPS) or the control (saline) added to the medium. The mRNA levels of the inflammatory cytokine genes, *Tnfa*, *Mcp1*, and *Il6* in the mouse primary hepatocytes were determined 4 h after stimulation by qRT-PCR*.* (**B**–**D**) Primary hepatocytes were pre-incubated for 20 min with glucose-free Krebs–Ringer bicarbonate buffer with or without 10 µM clodronate, followed by the administration of Krebs–Ringer buffer containing 25 mM glucose with or without 10 µM clodronate for the indicated time. The amounts of ATP (**B**) and triglycerides (**C**) in the culture supernatants, as well as the triglyceride contents in the primary hepatocytes (**D**), were evaluated at the indicated time points. The change in triglyceride content (**D**) was calculated based on the increase from the baseline value (hepatocytes treated with Krebs–Ringer buffer containing 25 mM glucose without clodronate at the time point of 10 min). (**E**) The mRNA expression levels of *Scd1*, *Srebp1c*, and *Cpt1a* were analyzed by qRT-PCR 60 min after either 0 mM or 25 mM glucose stimulation. Gene expression is shown as the fold change relative to hepatocytes treated with glucose-free Krebs–Ringer buffer. All data are shown as the mean ± S.E.M. *P < 0.05, **P < 0.01, ***P < 0.001. *NS* not significant.

Given the implications of crosstalk between immune cells and hepatocytes in the pathology of liver inflammation, we examined whether clodronate could suppress cytokine expression in hepatocytes as it does in macrophages in vitro^16^ . We exposed mouse primary hepatocytes to LPS and observed a strong increase in the mRNA expression of *Tnfa*, *Mcp1*, and *Il6* based on qRT-PCR. However, pre-treatment with clodronate could not prevent these increases in cytokine mRNA expression in the primary hepatocytes (Fig. 7A). Because glucose is reported to induce lipogenic gene expression and triglyceride accumulation in primary hepatocytes as in an overfeeding situation in vivo^27^, we exposed mouse primary hepatocytes to Krebs–Ringer buffer containing high glucose concentrations with or without 10 µM clodronate. We observed the early release of ATP from hepatocytes after glucose stimulation, and this release was significantly inhibited by the administration of clodronate (Fig. 7B). Consistent with the in vivo findings, clodronate also inhibited the glucose-induced secretion and accumulation of triglycerides in primary hepatocytes (Fig. 7C,D). In this in vitro model, however, the mRNA expression of *Scd1*, *Srebp1c*, and *Cpt1a* was not affected by stimulation with glucose or treatment with clodronate (Fig. 7E).

## Discussion

It has been proposed that extracellular nucleotides serve as “metabolokines” that link inflammation and metabolic processes in the liver^5^. Therefore, the functions of purinoceptors in various cellular components of the liver have been widely investigated. However, the importance of extracellular ATP release pathways in liver pathology was less understood. In this study, we provided compelling evidence that vesicular ATP release via VNUT plays a pivotal role in acute and chronic inflammation, as well as lipid metabolism in the liver, and we show that VNUT represents a potential therapeutic target for liver diseases.

In our GalN/LPS-induced liver injury model, clodronate reduced hepatic neutrophil infiltration, the apoptosis of hepatocytes, and inflammatory cytokine production. This experimental animal model recapitulates the clinical scenario of fulminant hepatic failure in humans^28^. Low doses of LPS in combination with the specific hepatotoxic agent, D-GalN, which sensitizes hepatocytes to the lethal effects of LPS, induce the production of inflammatory cytokines such as TNFα, IL-β, and IL-6, particularly in the macrophages, thereby promoting the infiltration of immune cells into the liver. Purinergic signaling is known to regulate the chemotaxis, proliferation, differentiation, and release of inflammatory mediators of immune cells^29^. We previously reported that clodronate completely inhibits vesicular ATP release from THP1, a human monocytic cell line, and that the deletion of extracellular ATP blocks TNFα release from THP1 cells induced by LPS administration^16^. Recently, neutrophils were also reported to express VNUT in their secretory granules, which mediates vesicular ATP release to promote neutrophil migration^25^. In the present study, we demonstrated that clodronate also suppresses the secretion of ATP from hepatocytes. Our results suggest that VNUT plays an important role in the recruitment of immune cells to the liver and in the production of cytokines by these cells under acute inflammation.

The mechanisms underlying NASH development are multifactorial and comprise insulin resistance, nutritional factors, adipose tissue dysfunction, and the activation of inflammatory pathways^30^. We previously found that glucose-induced ATP secretion does not occur in the hepatocytes of *Vnut*^*−/−*^ mice^20^, and ATP secretion was inhibited by clodronate in the present study. These findings suggest that VNUT links nutritional status to the initiation of inflammation. In addition, extracellular ATP causes insulin resistance in the liver^31^. Indeed, we previously found that *Vnut*^−/−^ mice exhibit increased insulin sensitivity of the liver^15^. In the current study, we observed that the administration of clodronate reduced steatosis and macrophage infiltration in both MCD diet-induced and HFHC diet-induced NASH models. In NASH, insulin resistance predisposes the liver to fat accumulation and causes lipotoxicity, which induces hepatocellular apoptosis and the recruitment of immune cells, while the activation of pro-inflammatory pathways maintains insulin resistance in turn^32^. By inhibiting vesicular ATP release both from the hepatocytes and immune cells, clodronate is expected to interrupt this vicious cycle in the microenvironment of the liver.

Notably, clodronate prevented the development of fibrosis in diet-induced NASH models. Purinergic signaling is implicated in the pathogenesis of hepatic fibrosis^33^. Adenosine acts on A2A receptors to increase collagen production by hepatic stellate cells^34^. The treatment of human hepatic stellate cells with a P2X7 receptor antagonist is reported to suppress the activation of stellate cells induced by LPS or the conditioned medium from LPS-stimulated mouse macrophages^35^. Therefore, it is suggested that clodronate prevents the progression of liver fibrosis by directly preventing stellate cell activation and/or by affecting their cross-talk with macrophages. The blockade of vesicular ATP release by clodronate could be a potential therapeutic option for the treatment of liver fibrosis.

Concerning hepatic lipid metabolism, the genetic depletion of the P2X7 receptor was reported to reduce hepatic fat accumulation and lipogenesis-related gene expression induced by a high-fat (HF) diet^8^. In our study, the oral administration of clodronate significantly suppressed the expression of de novo FA synthesis-related genes including *Scd1*, *Acc*, and *Srebp1c* in the liver of NC diet-fed mice. Given that the liver-specific knockout of *Acc* or *Scd1* was shown to protect mice from steatosis^36–38^, the attenuation of steatosis by clodronate may be the consequence of the suppression of these genes. However, after 24 weeks of HFHC diet feeding, these de novo FA synthesis-related genes were strongly downregulated regardless of clodronate treatment. While the genes involved in triglyceride synthesis (*Dgat2*) and VLDL secretion (*Apoa5*) were relatively upregulated by clodronate in HFHC diet-fed mice, the hepatic lipid content was reduced and the serum triglyceride and total cholesterol levels were unchanged in mice treated with clodronate compared with the control group. This suggests that changes in the expression of these genes could form part of a compensatory mechanism.

In addition to being directly affected by purinergic signaling as shown with our primary cultured hepatocytes^20^, the development of steatosis could also have been influenced by inflammatory mediators in adipose tissue that induce systemic insulin resistance. Although the protein levels of IL1β, TNFα, and MCP1 in the plasma were not altered in our experimental NASH models following the administration of clodronate, an effect of the changes in the local inflammatory condition of the adipose tissue could not be excluded. The increased systemic and hepatic insulin sensitivity in VNUT-knockout mice also support this hypothesis^15^.

Clodronate can be intracellularly metabolized to an analog of ATP (AppCCl_2_p), which inhibits the mitochondrial ADP/ATP translocase^39^ and various other kinases such as PDGFRa, JAK2, JAK3, and FGFR2^40^. These pharmacological properties of clodronate may also explain the suppression of steatosis. However, we recently demonstrated that VNUT-knockout mice are also protected from NASH^20^. In this earlier study, the inhibitory effect of clodronate on the triglyceride secretion from hepatocytes was canceled by 2-methylthio-ADP, indicating that clodronate suppresses triglyceride secretion by blocking purinergic signaling. While clodronate liposomes induce the apoptosis of macrophages by inhibiting mitochondrial ADP/ATP translocase, clodronate alone does not affect the viability of the human monocyte cell line, THP1^16^. Furthermore, clodronate did not increase but instead reduced cell apoptosis in our LPS/D-Gal-induced acute liver injury model. Taken together, the protective effect of clodronate against acute and chronic liver injury is expected to mainly depend on the VNUT–purinoceptor pathway. To better characterize the role of VNUT and the mechanism of action of clodronate in NASH, further investigations using the conditional knockout approach and detailed biochemical analysis will be required.

In our in vitro experiment with primary hepatocytes, clodronate inhibited both the secretion and accumulation of triglycerides without affecting the mRNA expression of *Scd1* and *Srebp1c*. These results indicated that triglyceride accumulation was inhibited by clodronate via mechanisms other than the transcriptional regulation of de novo FA synthesis genes. A recent study demonstrated that treating mice with *Apob* antisense oligonucleotides decreases the secretion of VLDL without causing hepatic steatosis^41^. Without apolipoprotein B (apoB), which is essential for the hepatic assembly and secretion of triglyceride-rich VLDL, triglycerides become trapped in the lumen of the endoplasmic reticulum (ER). This triggers ER autophagy followed by the oxidation of the released FA, which, in turn, prevents steatosis. ADP was reported to stimulate apoB secretion through P2Y13 receptors^31^. We recently demonstrated that VNUT is partially colocalized with APOB in hepatocytes and that glucose-induced triglyceride secretion from hepatocytes is blocked by a P2Y13 inhibitor^20^. Taken together, it is possible that clodronate reduces both the secretion and accumulation of triglycerides by inhibiting P2Y13 signaling and apoB activity. Further studies will be required to clarify the role of purinergic signaling in the regulation of autophagy and lipid metabolism.

In summary, our study revealed that clodronate attenuated hepatic inflammation, fibrosis, and steatosis. The pharmacological inhibition of VNUT may represent a potential therapeutic approach for the treatment of hepatic inflammatory and metabolic diseases.

## Methods

### Animal experiments

C57BL/6 wild-type mice were obtained from Charles River Laboratories Japan, Inc. Control mice were fed ad libitum with a normal chow (NC) diet (5.4% fat, CRF-1; Orient Yeast Co.) and kept under a 12-h light–dark cycle. The MCD group of mice was fed a methionine- and choline-deficient diet (A02082002B; Research Diets) and the HFHC group was fed a high-fat, high-cholesterol diet (D09100301; Research Diets) that was enriched in fat (40% kcal including premix shortening), fructose (22% by weight), and cholesterol (2% by weight). Animals were allowed ad libitum access to these diets for the indicated periods. Mice fed the MCD diet received a daily subcutaneous dose of 20 mg/kg clodronate or the vehicle. Mice fed an HFHC diet were given 30 mg/kg clodronate daily or the vehicle alone in their drinking water.

For the analysis of acute liver injury, 10-week-old male mice received intraperitoneal (i.p.) injections of 700 mg/kg d-galactosamine (GalN) and 100 µg/kg lipopolysaccharide (LPS; *E. coli* 0111:B4). Mice were pretreated with saline or 50 mg/kg i.p. clodronate 1 h before GalN and LPS administration.

All experiments were carried out in accordance with the approved institutional guidelines and were approved by the Ethics Committees of Kyushu University, Graduate School of Medicine (approval number: A29-138-0) and the Kurume University School of Medicine (approval number: 2020-115).

### Histology

Liver samples were fixed (4% paraformaldehyde), paraffin-embedded, sectioned, and stained with hematoxylin–eosin or Picrosirius Red. Immunostaining was performed on paraffin-embedded sections using the F4/80 monoclonal antibody (1:200, Cat# MCA497GA; AbD Serotec) or Ly6G monoclonal antibody (1:200, Cat# 551459; BD Biosciences). TUNEL staining was performed with the ApopTag Peroxidase In Situ Apoptosis Detection Kit (Cat# S7100; Millipore). The histological score was determined by a blinded investigator using the Nonalcoholic Steatohepatitis Clinical Research Network histological scoring system for NAFLD^42^. The total NAFLD activity score (NAS) was calculated from the sum of the individual scores for steatosis, lobular inflammation, and hepatocellular ballooning. Each parameter ranged from 0 to 2 (ballooning) or 3 (the other two scores), with 0 being normal and 2 or 3 being severe. Fibrosis staging was based on the NASH fibrosis stage and ranged from 0 to 4^42^. The numbers of F4/80- or Ly6G-positive cells were counted in four high-power fields per section using the BZ-X Hybrid Cell Count software (Keyence). The numbers of TUNEL-positive cells were counted in 16 high-power fields per section.

### Biochemical assays

The levels of alanine aminotransferase (ALT) in the plasma were measured using a DRI-CHEM 3500 Chemistry Analyzer with the DRI-CHEM slide GPT/ALT-P III (Fujifilm). Serum osteopontin was analyzed with a mouse osteopontin assay kit (Immuno-Biological Laboratories). The total liver lipid content was determined with Folch’s method^43^. Triglyceride, total cholesterol, and nonesterified fatty acid (NEFA) levels were measured with the TG E-test, Cholesterol E-test, and NEFA C-test (Wako), respectively. The levels of IL1β, TNFα, IL6, and MCP1 in the plasma and liver lysate were analyzed using the BD Cytometric Bead Array (BD Biosciences) according to the manufacturer’s instructions. A NovoCyte flow cytometer (ACEA Biosciences) was used to quantify the cytokine profiles. ATP was determined with the Kinsiro ATP Luminescence Kit (Toyo B-Net).

### mRNA analyses

Total RNA was isolated from the mouse liver and primary hepatocytes using TRIzol Reagent (Invitrogen). Reverse transcription with 1 µg of RNA was conducted using the QuantiTect Reverse Transcription Kit (Qiagen). Quantitative real-time PCR was used to determine the relative expression levels of mRNA. The assays were performed with SYBR Premix Ex Taq II (Takara Bio) on the Applied Biosystems 7500 Real-Time PCR system. The primer sequences of the selected genes are provided in Supplementary Table S1 online. Results were normalized to the expression of glyceraldehyde 3-phosphate dehydrogenase (Gapdh) and are shown as the fold change relative to gene expression in the control mice or hepatocytes.

### Primary culture of mouse hepatocytes

Primary hepatocytes were isolated from 10-week-old C57BL/6 male mice as previously described^44^. Hepatocytes were seeded in 6-well, collagen-coated culture dishes at 2 × 10^6^ cells per well in Dulbecco’s modified Eagle’s medium (Sigma-Aldrich) supplemented with 1 μM insulin, 2 mM l-glutamine, 10 IU/mL penicillin, 10 IU/mL streptomycin, and 10% fetal bovine serum. For the analysis of cytokine gene expression in hepatocytes, primary mouse hepatocytes were washed three times and pre-incubated for 30 min with Dulbecco’s modified Eagle’s medium supplemented with 10% fetal bovine serum, followed by stimulation with 1 µg/mL LPS or control medium. The pre-incubation and stimulation steps were performed in the presence or absence of 10 µM clodronate. At 4 h after stimulation, the hepatocytes were collected for mRNA analysis. For the analysis of ATP and triglyceride levels, primary hepatocytes were washed three times and pre-incubated for 20 min with glucose-free Krebs–Ringer bicarbonate buffer (10 mM HEPES-Tris, pH 7.4, 128 mM NaCl, 1.9 mM KCl, 1.2 mM KH_2_PO_4_, 1.3 mM MgSO_4_, 26 mM NaHCO_3_, 2.4 mM CaCl_2_, 0.2% (w/v) bovine serum albumin, and 300 μM oleic acid) with or without 10 µM clodronate. After pre-incubation, the hepatocytes were stimulated with Krebs–Ringer buffer containing 25 mM glucose with or without 10 µM clodronate for the indicated time. The culture supernatants and cell lysates were then subjected to biochemical analysis. For the analysis of lipid gene expression, the hepatocytes were collected at 60 min after stimulation. All experiments were performed in duplicate at least three times.

### Statistical analysis

All results are reported as the means ± standard error of the mean (S.E.M.). Statistical analyses were performed using GraphPad Prism 7.0 software (GraphPad Software). The unpaired two-tailed Student’s *t* test was used to assess significance when comparing two groups. Statistical significance between three or more groups was determined using two-way analysis of variance (ANOVA) with Tukey’s or Dunnett’s post hoc test. Differences were considered statistically significant when the *P* value was < 0.05.

## Supplementary Information

Supplementary Information

## Data Availability

The datasets generated during the current study are available from the corresponding author on reasonable request.
