# Supplementary material for: Clodronate, an inhibitor of the vesicular nucleotide transporter, ameliorates steatohepatitis and acute liver injury
Source: Sci Rep. 2021 Mar 4;11:5192. doi: 10.1038/s41598-021-83144-w (PMC7933178; doi:10.1038/s41598-021-83144-w)
Supplement: Supplementary file 1 — Supplementary Information [file 41598_2021_83144_MOESM1_ESM.pdf]

Supplementary Information

Clodronate, an inhibitor of the vesicular nucleotide transporter, ameliorates steatohepatitis and acute liver injury

Nao Hasuzawa[1,2,*], Keita Tatsushima[2,3], Lixiang Wang[4], Masaharu Kabashima[1], Rie Tokubuchi[1], Ayako Nagayama[1], Kenji Ashida[1], Yoshihiro Ogawa[2], Yoshinori Moriyama[1], and Masatoshi Nomura[1]

[1]Division of Endocrinology and Metabolism, Department of Internal medicine, Kurume University School of Medicine, Kurume 830-0011, Japan

[2]Department of Medicine and Bioregulatory Science, Graduate School of Medical Sciences, Kyushu University, Fukuoka 812-8582, Japan

[3]Department of Psychosomatic Medicine, Graduate School of Medical Sciences, Kyushu University, Fukuoka 812-8582, Japan

[4]Department of Chemistry, Kurume University School of Medicine, Kurume 830-0011, Japan

*Corresponding author:

Nao Hasuzawa

E-mail: hasuzawa@med.kurume-u.ac.jp

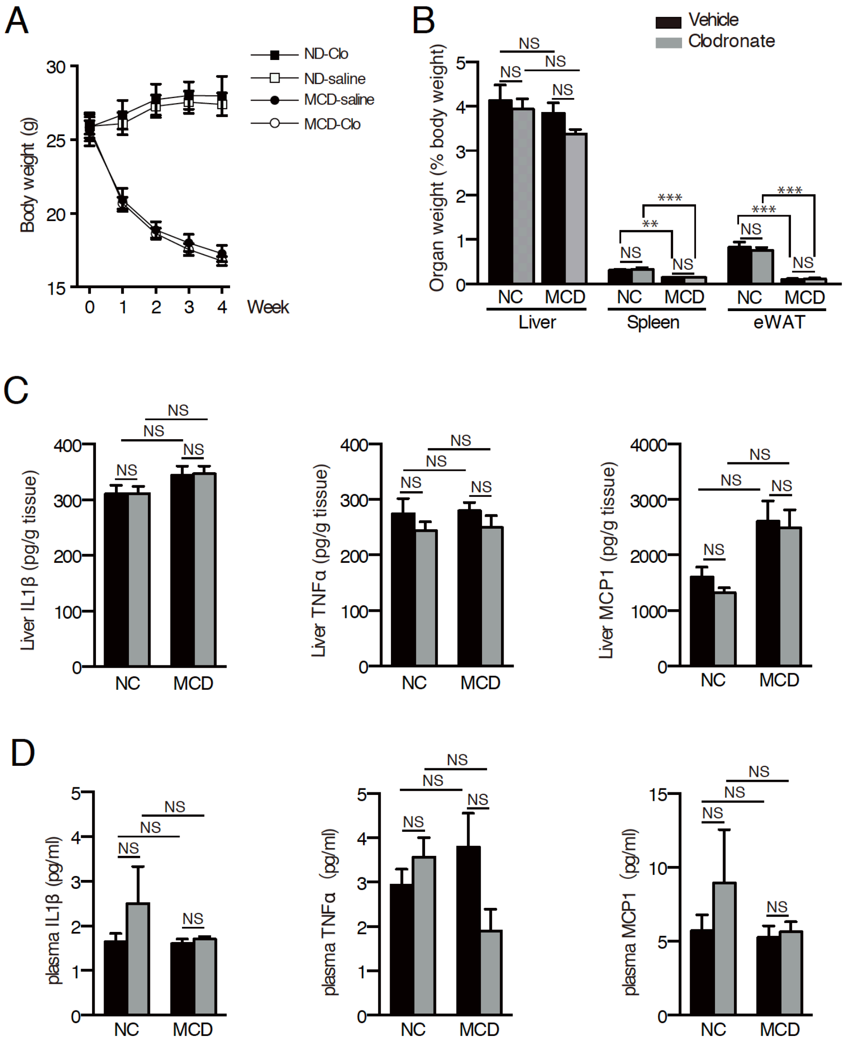

Supplementary Figure S1 Hasuzawa et al.

Supplementary Figure 1. Physiological measurement and inflammatory cytokine analysis of mice fed the methionine- and choline-deficient diet.

Mice treated as described in Fig. 1 were analyzed (n = 5 MCD diet-fed, per treatment; n = 3–5 NC-fed, per treatment). (A) Bodyweight change. (B) Organ weight expressed as a percentage of the bodyweight at the end of the study. (C) Protein contents of IL1β, TNF, and MCP1 in the liver as determined by BD Cytometric Bead Array. All data are shown as the mean ± SEM. *$P < 0.05$, **$P < 0.01$, ***$P < 0.001$. NS, not significant; NC, normal chow; MCD, methionine- and choline-deficient diet.

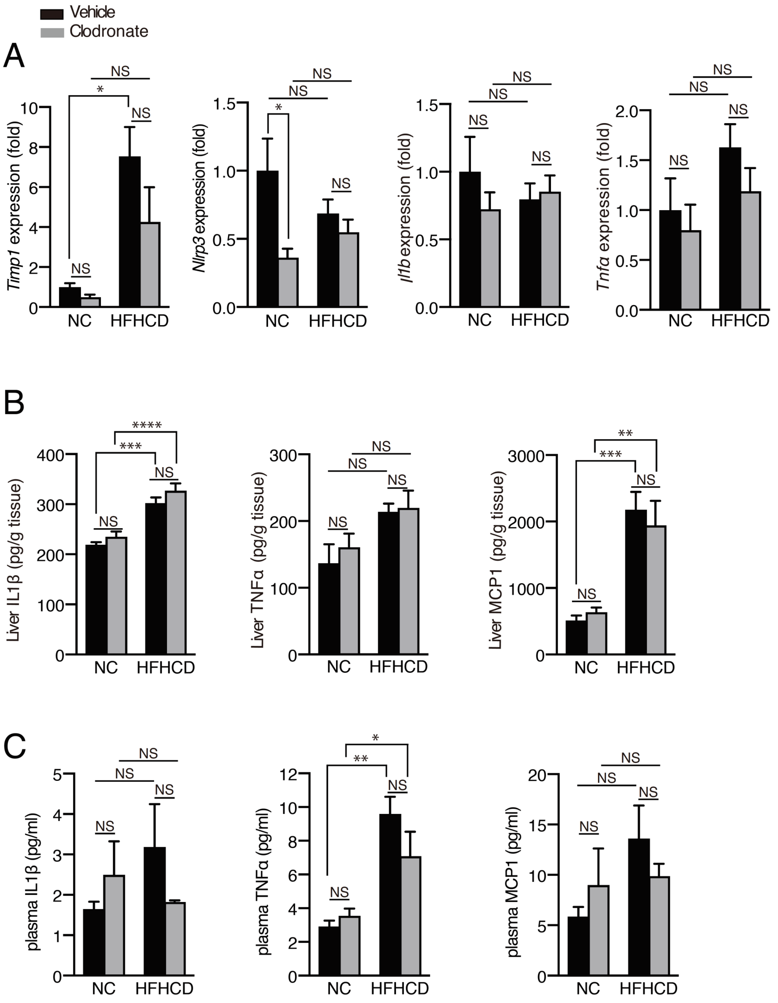

Supplementary Figure S2 Hasuzawa et al.

Supplementary Figure 2. Inflammatory cytokine analysis of the liver of high-fat, high-cholesterol diet-fed mice.

The hepatic inflammatory cytokines of mice treated as described in Fig. 3 were investigated (n = 5 per treatment group). The hepatic mRNA levels of the inflammatory genes (A) *Timp1*, (B) *Nlrp3*, (C) *Il1β*, *and* (D) *Tnfa* were determined by qRT-PCR, while the hepatic protein contents of (E) IL1β, (F) TNF, and (G) MCP1 were determined by the BD Cytometric Bead Array. All data are shown as the mean ± S.E.M. **P* < 0.05, ***P* < 0.01, ****P* < 0.001. NS, not significant; NC, normal chow; HFHCD, high-fat and high-cholesterol diet.

Supplementary Table S1. Primer sets for RT-PCR

Gene	Forward primer (5'-3')	Reverse primer (5'-3')	Product size(bp)
Nlrp3	GATCTTTGCTGCGATCAACA	CAGCAAACCCATCCACTCTT	139
Il-1b	TGTGAAATGCCACCTTTTGA	GGTCAAAGGTTTGGAAGCAG	94
Tnf	CCACCACGCTCTTCTGTCTA	AGGGTCTGGGCCATAGAACT	103
Il-6	CCGGAGAGGAGACTTCACAG	TTCTGCAAGTGCATCATCGT	166
F4/80	TGACAACCAGACGGCTTGTG	GCAGGCGAGGAAAAGATAGTGT	61
Mcp1	CCCAATGAGTAGGCTGGAGA	TCTGGACCCATTCCTTCTTG	125
Timp1	GTGGGAAATGCCGCAGAT	GGGCATATCCACAGAGGCTTT	67
Col1a1	GCGGTTCAGGTCCAATGGGT	GTTCCAGGCAATCCACGAGC	139
Scd1	AGTGCCGCGCATCTCTATG	AAGGGGAAGGTGTGGTGGT	122
Acc	CCAGGCCATGTTGAGACGCT	ATCACAGAGCGGACGCCATC	132
Srebp1c	GATCAAAGAGGAGCCAGTGC	TAGATGGTGGCTGCTGAGTG	191
Dgat2	TACTTCACCTGGCTGGCATT	GTGGTCAGCAGGTTGTGTGT	143
Apoa5	AGGCAGCAGTTGAAACCCTA	TGAGCCTTGGTGTCTTCTCC	107
Mttp	TGAGCGGCTATACAAGCTCAC	CTGGAAGATGCTCTTCTCGC	220
Ppara	TGCCGTGTGAGACCCTGTTT	AGGAGGGAGTGGGGAAGGTC	159
Cpt1a	CCAGGCTACAGTGGGACATT	GAACTTGCCCATGTCCTTGT	209
